# Automated segmentation of midbrain nuclei using deep learning and multisequence MRI: A longitudinal study on iron accumulation with age

**DOI:** 10.1162/imag_a_00304

**Published:** 2024-10-07

**Authors:** Farshad Falahati, Jonatan Gustavsson, Grégoria Kalpouzos

**Affiliations:** Aging Research Center, Department of Neurobiology, Care Sciences and Society, Karolinska Institutet and Stockholm University, Stockholm, Sweden

**Keywords:** midbrain, quantitative susceptibility mapping, automated segmentation, deep learning, longitudinal, aging

## Abstract

Elevated levels of brain iron, particularly within the basal ganglia, have been associated with cognitive and motor impairment in normal aging and neurodegenerative conditions. The subthalamic nucleus (STN), substantia nigra (SN), and red nucleus (RN), despite their high iron content and contribution to motor and cognitive processes, are less frequently studied. This oversight can largely be attributed to the challenges posed by in-vivo assessments of these small, deep-seated midbrain structures. We developed and validated an automated tool for the segmentation of the STN, SN, and RN. Multi-sequence magnetic resonance imaging (MRI) data, including T1-weighted, FLAIR, Quantitative Susceptibility Mapping (QSM) and$R_{2}^{*}$alongside manual delineation on QSM images of 40 individuals, were used to train segmentation models based on the nnU-Net deep-learning framework. A combination of QSM and FLAIR sequences was found to be optimal for structure segmentation (mean Dice scores of 0.84, 0.91, and 0.94 for STN, SN, and RN, respectively). We next applied the automated segmentation method to an independent 3-year longitudinal dataset, including 175 healthy adults (age range at baseline: 20–79 years old). Structural equation modelling was used to assess iron accumulation over time using age, sex, baseline iron, and regional volume as factors of interest. Cross-sectionally, older age was linearly associated with higher iron load in SN and STN; the association was non-linear in RN. Longitudinally, results indicated significant iron accumulation in the STN (Mean increase = 0.02,*p*= 0.005) and SN (Mean increase = 0.035,*p*= 0.001), but not in the RN (Mean increase = 0.015,*p*= 0.2). Our findings demonstrated high performance of nnU-Net in automated segmentation, and advanced our understanding of iron accumulation in midbrain nuclei in aging.

## 1 Introduction

Aging is accompanied by brain deterioration, manifesting as cognitive and motor decline (Cabeza et al., 2016;Fjell et al., 2014;Grady, 2012;Nyberg et al., 2012). Iron is a mineral essential for numerous biological functions in the brain such as oxygen transportation, DNA replication, mitochondrial respiration, neurotransmitter synthesis, and myelination (Ke & Qian, 2007;Mills et al., 2010;Moos et al., 2007;Todorich et al., 2009). Yet, iron overload is toxic as it may trigger oxidative stress and inflammation of brain tissue (Haider et al., 2014;Ward et al., 2014;Winterbourn, 1995;Zecca et al., 2004). An elevated level of brain iron has been observed not only in neurodegenerative diseases such as Alzheimer’s disease (AD) and Parkinson’s disease (PD) but also during normal aging, especially in basal ganglia (Burgetova et al., 2021;Dusek et al., 2022;Y. Zhang et al., 2018a). A link between elevated brain iron and reduced cognitive and motor performance in aging has been reported in several studies (Kalpouzos et al., 2017;Madden & Merenstein, 2023;Penke et al., 2012;Rodrigue et al., 2020;Salami et al., 2018,^2021^;Spence et al., 2020).

While prominent iron-rich regions like striatum have been explored in aging research, smaller brain structures like the substantia nigra (SN), subthalamic nucleus (STN), and red nucleus (RN) are often overlooked, despite their significant iron content and involvement in motor and cognitive functions (Boecker et al., 2008;Dahl et al., 2023). In PD, higher iron load in the SN, STN, and RN has been related to severity of motor symptoms (Huang et al., 2020;Lewis et al., 2013;Tambasco et al., 2019;Ulla et al., 2013). However, there is limited knowledge regarding the integrity of these nuclei during the normal aging process. In previous studies, higher iron content in these regions was observed in older adults compared to their younger counterparts (Garzón et al., 2018; see alsoDaugherty & Raz, 2013for a meta-analysis). In these studies, however, the sample size was relatively small, and the design was cross-sectional. While cross-sectional designs may hint at mean age trends, longitudinal studies are crucial to reveal the true trajectories of age-related change in iron content (A. Daugherty & Raz, 2013). Also, sex differences in iron load have rarely been systematically investigated and existing studies provide contrasting insight (Hagemeier et al., 2013;Persson et al., 2015).

The limited study of the SN, STN, and RN can be attributed to technical challenges associated with in vivo assessment such as limited spatial resolution, low contrast, and the lack of efficient segmentation methods. Whereas manual delineation is deemed the “gold standard” for anatomical segmentation, it is precise but also time-consuming, subjective, and requires expert involvement. In contrast, automated segmentation methods promise greater efficiency, consistency, and scalability than their manual counterparts. Such methods provide objective analysis, and mesh well with advanced technologies, permitting swift processing of extensive datasets and allowing researchers and clinicians to prioritise result interpretation.

Typical automated segmentation methods that register a histological atlas to a subject’s anatomy (primarily using structural T1-weighted MR images) may encounter difficulties due to the low contrast in the deep gray matter nuclei of the midbrain (He et al., 2015). Segmentation performance can be enhanced by incorporating additional MRI contrasts, such as T2*-weighted and T2-weighted images, where the nuclei appear hypointense due to iron deposition (Garzón et al., 2018;Xiao et al., 2014). Quantitative susceptibility mapping (QSM) is a technique that estimates in-vivo tissue magnetic susceptibility distribution by solving the magnetic field-to-susceptibility source inverse problem using gradient echo phase images (de Rochefort et al., 2010). This technique offers a novel tissue contrast, making the magnetic susceptibility effects of various tissue components clearly visible. In particular, QSM images provide a superior contrast-to-noise ratio in certain brain structures, such as deep gray matter regions, which contain high paramagnetic iron content. In fact, the SN, STN, and RN are distinctly visible on QSM images, where the true lenticular shape of the STN is visible, allowing for its differentiation from the SN.

Atlas-based contouring algorithms, along with machine-learning techniques based on extracting hand-crafted features, have been dominant approaches for automated segmentation. Deep learning, a subset of machine learning, is quickly becoming the state-of-the-art method for image segmentation, owing to recent significant developments in computational capabilities and methodological refinements (Litjens et al., 2017). Deep learning consists of networks of artificial neurons with many layers, enabling learning from large amounts of data in a manner inspired by human brain function. Deep learning-based approaches have been applied to medical image segmentation tasks, achieving superior performance compared to traditional machine learning methods (Hesamian et al., 2019).

Convolutional Neural Networks (CNNs), a class of deep neural networks originally designed for image analysis, have been successfully utilised in a wide range of brain segmentation applications. These include the segmentation of brain tumors (Menze et al., 2015), tissue (Moeskops et al., 2016), and regions such as striatum (Choi & Jin, 2016), thalamus (Dolz et al., 2018), hippocampus (Ataloglou et al., 2019), and hippocampal subfields (Manjón et al., 2022). Although these applications showcase the versatility and effectiveness of CNNs in neuroimaging, only a few studies have explored their use in segmenting midbrain nuclei (Beliveau et al., 2021;Chai et al., 2022;Zhao et al., 2022). This shortage may be attributed to the challenges in obtaining sufficiently large and high-quality datasets, especially those with labelled QSM images, suggesting it as an area with potential for further in-depth exploration.

U-Net is a CNN-based architecture designed for fast and precise biomedical image segmentation (Ronneberger et al., 2015). The U-Net architecture consists of a contracting path (encoder path) and a symmetrical expanding path (decoder path) along with skip connections that bridge corresponding layers of the same size between the contracting and expanding paths. This unique architecture allows the network to capture the context in the image while maintaining the structural integrity, thus facilitating efficient learning of both high-level features and fine-grained details. Skip connections in U-Net bridge the encoding and decoding portions of the network, ensuring that spatial information is retained across the entire image. These attributes enhance the network’s learning capacity and enable it to make more accurate predictions with fewer training samples.

This study seeks to bridge two central gaps. First, we propose an automated segmentation approach for demarcating STN, SN, and RN, employing a powerful framework based on the U-Net architecture, coupled with multisequence imaging data. Second, using the obtained results from the automated segmentation, we aim to gain knowledge on age-related iron accumulation over time in midbrain nuclei using a longitudinal dataset.

## 2 Materials and Methods

### 2.1 Study population

We used data from two research projects. The first project, the cross-sectional “Iron Project,” involved 22 younger (10 women, age range 26–42) and 18 older individuals (8 women, age range 65–77). The second project, the longitudinal “IronAge Project,” had an initial cohort of 208 adults (108 females, age range 20–79), of whom 135 returned for a follow-up assessment approximately 3 years later (mean days between scans: 1,003, SD: 81). Participants in both projects were healthy volunteers, that is, without a history of neurological or psychiatric diseases or conditions affecting iron metabolism (e.g., iron deficiency anemia, hemochromatosis). Each project received approval from the Regional Ethical Review Board in Stockholm, and all participants provided informed consent prior to data collection. Detailed descriptions of the Iron project are available inGarzón et al. (2018), whereas the IronAge project is detailed inKalpouzos et al. (2021)andGustavsson et al. (2022).

### 2.2 Imaging data

#### 2.2.1 MRI data acquisition

In both the Iron and IronAge projects, participants were scanned using the same GE Discovery MR750 3.0T scanner, equipped with an 8-channel phased array receiving coil, at the MR center of Karolinska University Hospital in Stockholm, Sweden. The following structural MRI sequences were obtained in both projects and used for this study:

A*3D T1-weighted IR-SPGR*sequence with parameters: repetition time (TR) = 6.96 ms, inversion time (TI) = 450 ms, echo time (TE) = 2.62 ms, flip angle = 12°, field of view (FOV) = 24 cm, reconstructed into 176 axial slices with a 1 mm thickness, and an in-plane resolution of 0.94 mm x 0.94 mm.

A*3D T2-weighted FLAIR*sequence with parameters: TR = 8,000 ms, TI = 2,259 ms, effective TE = 114 ms, echo train length 180 echoes, flip angle = 90°, FOV = 27 cm, reconstructed into 292 sagittal slices with a 0.6 mm thickness, and an in-plane resolution of 0.53 mm x 0.53 mm.

*A 3D multi-echo gradient recalled echo (meGRE)*sequence was acquired for all participants in the Iron project, as well as for 175 participants at baseline and 134 participants at follow-up in the IronAge project, with 119 subjects having both baseline and follow-up data. Parameters were: TR = 37.52 ms, 8 echo times (first TE = 3.74 ms, followed by 7 consecutive echoes with a constant echo spacing of 4.31 ms), flip angle = 20°, FOV = 24 cm, reconstructed into 146 axial slices with a 1 mm thickness, and an in-plane resolution of 0.94 mm x 0.94 mm.

#### 2.2.2 Quantitative susceptibility mapping

Morphology-enabled dipole inversion (MEDI), as introduced byLiu, Liu, et al. (2011), is a refined approach to reconstruct the susceptibility map from MRI phase data. This technique addresses the complex inversion problem inherent in QSM, where one aims to discern the underlying tissue susceptibility causing the observed phase. MEDI incorporates the spatial structure or morphology of the underlying tissues into the inversion process, thereby adding constraints that enhance the robustness of the solution. By using L1-norm regularization, which promotes sparsity, MEDI produces sharper susceptibility maps with reduced streaking artifacts, a common issue in traditional QSM reconstructions.

Here, we employed the recommended nonlinear variant of MEDI (Liu et al., 2013) and implemented in the MEDI Toolbox (http://weill.cornell.edu/mri/pages/qsm.html), to calculate QSM images for all meGRE images in both the Iron and IronAge datasets. Initially, the total field map was estimated from the complex meGRE images by performing a nonlinear least square fitting on a voxel-by-voxel basis. The resulting frequency map was then spatially unwrapped using a guided region-growing unwrapping algorithm (W. Xu & Cumming, 1999). The background fields, that is, the superimposed field contributions that are not caused by the sources inside the brain and mainly generated by air-tissue interferences, were eliminated using a nonparametric technique based on projection onto dipole fields (Liu, Khalidov, et al., 2011). Finally, the corrected frequency map was used as input for the field-to-source inverse problem to calculate the map of susceptibility in parts per million (ppm) units.

Due to the singularity of dipole kernel at the center of k-space, the generated QSM images contain relative susceptibility values. Therefore, the QSM images may not necessarily be comparable across subjects in a cohort. A typical approach to address this issue is zero-referencing where a common tissue is chosen as a reference and its average susceptibility is subtracted from the susceptibility values of other tissues. In this study, a region within the cortical white matter was selected as the reference region. The coordinates of the center of this region in MNI space in neurological convention were [-24, -27, 38], labelled as the corticospinal tract according to the NatBrainLab atlas of white matter (Catani & Thiebaut de Schotten, 2008). To perform zero-referencing, we initially generated unreferenced QSM images using the MEDI toolbox for each participant, as described above. Next, the selected coordinate in MNI space was mapped first to individual space using a non-linear registration warp. Then, using an in-house developed region-growing algorithm, a region of 1,000 voxels centred on the mapped coordinate was created. The algorithm uses the white-matter mask to ensure the created region encompassed white-matter tissue only. The FMRIB Software Library (FSL,http://fsl.fmrib.ox.ac.uk) was used to calculate non-linear transformation parameters (Andersson et al., 2007) and to obtain the white-matter mask (Y. Zhang et al., 2001). Finally, we calculated the reference value from white matter, which was then subtracted from the unreferenced QSM images to generate zero-referenced QSM images. This approach was previously verified in both Iron (Garzón et al., 2017) and IronAge (Gustavsson et al., 2022) studies.

#### 2.2.3 Relaxometry

A quantitative map of the effective transverse relaxation rate,$R_{2}^{*}$, was generated by fitting an exponential decay model to each voxel of the magnitude component of meGRE images, according to:

$$S^{2}=S_{0}^{2}.exp\left(-2.TE.R_{2}^{\text{*}}\right)$$ [1]

where S is the measured signal magnitude, TE is the echo time, and S_0_is the signal intensity at TE = 0 (Deistung et al., 2013;Miller & Joseph, 1993).

#### 2.2.4 MRI data co-registration

To align all MRI data within a common spatial framework, for each subject, the QSM,$R_{2}^{*}$maps, and FLAIR images were registered to subject’s T1 image. For each meGRE sequence, a representative image was produced by computing the root mean square of the magnitude images from the first four echoes. This allowed for the calculation of the transformation matrices from meGRE spaces to T1 space. To ensure uniformity in data treatment and analysis, the same MRI data processing pipeline was applied to both the Iron and IronAge datasets.

#### 2.2.5 Manual tracing

The SN, STN, and RN of each hemisphere were manually delineated by G.K. on QSM images for all 40 subjects of the Iron project, using the MRIcron software (https://github.com/neurolabusc/MRIcron). The manual tracing was conducted twice on separate occasions in the whole sample to ensure intra-rater reliability. The average Dice coefficient derived from these repeated tracings was 0.81, with specific values of 0.75, 0.82, and 0.88 for the STN, SN, and RN respectively, indicating strong agreement and suggesting that the tracings were reliably reproducible (seesection 2.3.4for the definition and calculation of Dice score). The Duvernoy’s Atlas of the Human Brain Stem and Cerebellum (Naidich et al., 2009) was used as anatomical reference, and the contrast in susceptibility images aided to determine structure boundaries in the 3 anatomical planes (Garzón et al., 2018).

### 2.3 Segmentation

#### 2.3.1 Experimental setup

Initially, the efficacy of the deep learning-based segmentation technique was assessed, with an emphasis on determining the influence of various input sequences on performance. Deep learning models were individually trained using a single-sequence approach with the following images as input: QSM,$R_{2}^{*}$, FLAIR, and T1-weighted. Next, a multi-sequence regimen was adopted where combinations of the aforementioned sequences were used as inputs. The Iron dataset was utilised for these preliminary evaluations. A 20–80 split was applied to divide the dataset into testing and training subsets. Eight subjects were randomly selected to form the testing cohort, while the remaining 32 subjects were used for training. A five-fold cross-validation strategy was employed during training to ensure a thorough evaluation of the model’s robustness across different subsets of the data. This approach aids not only in mitigating potential overfitting but also in evaluating the model’s ability to generalise and perform effectively on new, unseen data (Falahati et al., 2014).

Based on the initial training and evaluation, the model that exhibited optimal performance with specific input modality combinations was identified. This model was subsequently refined using the complete Iron dataset, encompassing all 40 subjects. Following this refinement, the IronAge dataset was processed using the model.

#### 2.3.2 Deep learning model architecture

The nnU-Net (Isensee et al., 2021) is a self-adapting semantic segmentation method that has gained significant recognition in medical image segmentation tasks. Rooted in the foundational U-Net architecture, nnU-Net integrates self-adapting mechanisms to determine the optimal network configuration and hyperparameters based on the dataset’s characteristics. While the core design principles of nnU-Net, such as its symmetric encoder-decoder architecture, loss function, and optimiser, remain intact, nnU-Net automatically modifies the network’s configuration—including the network’s depth, batch size, and patch size—depending on the dataset’s characteristics. This adaptability ensures nnU-Net to avoid the pitfalls associated with excessive complexity, which could lead to overfitting, as well as those stemming from oversimplification, which might result in under-segmentation.

**Fig. 1.**
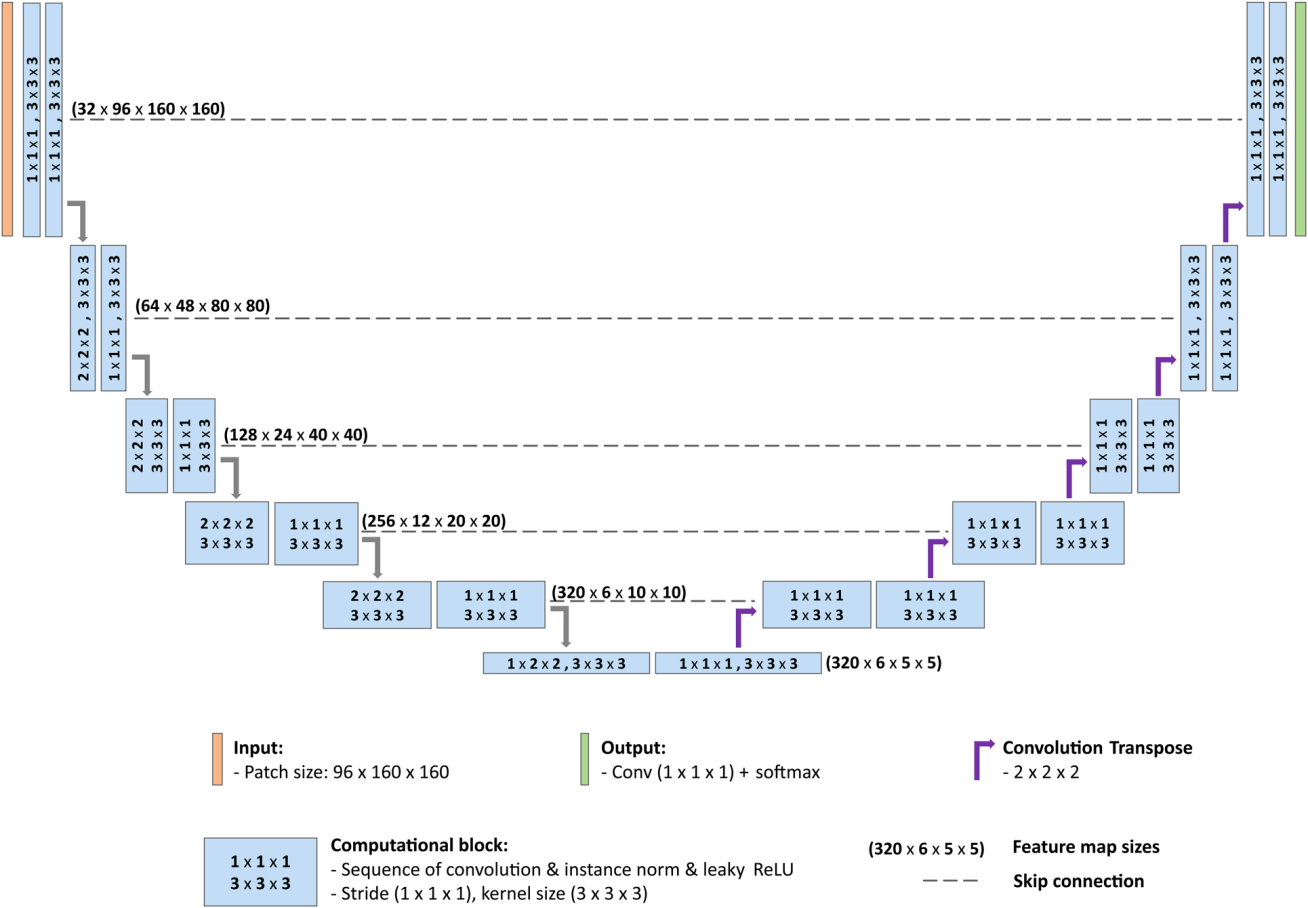
The architecture of nnU-Net deep learning network. The blue boxes represent computational blocks, each consisting of a series of plain convolutions, instance normalisation, and the Leaky ReLU activation. Within each box, the stride of the convolution is indicated by the first set of three numbers, whereas the kernel size of the convolution is denoted by the second set of numbers. Dashed lines depict skip connections. Numbers in parentheses indicate the feature map sizes at each resolution level.

For the segmentation of structures in this study, we employed the 3D full-resolution configuration of nnU-Net.Figure 1illustrates the model’s architecture, which features 11 resolution levels: 5 in the contracting path, a central bottleneck, and 5 in the expanding path. Each resolution level contains two computational blocks. These blocks are composed of a series of plain convolutions, followed by instance normalisation and the leaky rectified linear unit (Leaky ReLU) activation. All convolutional kernel sizes have a matrix dimension of 3 x 3 x 3. For downsampling, the model uses strided convolutions (all sized 2 x 2 x 2, except for the bottleneck which is 1 x 2 x 2). Upsampling is achieved using transposed convolutions. In nnU-Net, a larger patch size is prioritised over a larger batch size. Consequently, the model is configured with a patch size of 96 x 160 x 160 and a batch size of 2. All training sessions were set to run for a fixed length of 1,000 epochs, with each epoch defined as 250 training iterations.

#### 2.3.3 Data augmentation

To increase the size of the training samples, data augmentation using a variety of transformations was applied on the training data. Accommodating the volumetric nature of the data, data augmentation strategy involved rotations, elastic deformations, and intensity variations, ensuring that the model is robust against subject-specific variations. For each training epoch, all images were augmented, hence providing new random samples. In nnU-Net, the augmentations are categorised into spatial augmentations, encompassing rotation, scaling, and simulation of low resolution, and intensity augmentations, featuring zero-centred additive Gaussian noise, Gaussian blurring, brightness and contrast adjustments, and gamma augmentation.

#### 2.3.4 Performance metric

To assess the segmentation performance, the Dice similarity coefficient (Dice score) was utilised (Dice, 1945). This metric offers a quantitative measure of the spatial overlap between two structures. The Dice score is calculated according to:

$$Dice\;score\;=\;\;\frac{2\left|\text{X}\cap \text{Y}\right|}{\left|X\right|+\left|Y\right|}$$ [2]

where,XandYrepresent two different structures (e.g., manually delineated label and predicted segmentation by the model).|X∩Y|denotes the count of common elements betweenXandY,|X|and|Y|indicate the number of elements in each set. A Dice score of 1 signifies perfect overlap, whereas a score of 0 indicates no overlap.

### 2.4 Statistical analyses

The average iron values and volumes of the nuclei were extracted from QSM images, both from manually delineated labels and segmentations produced by cross-validated models. The deep learning model generated segmentation labels for the left and right hemispheres separately; however, for statistical analyses, we averaged the measures obtained from the left and right hemispheres. This approach was taken to prioritise global aging patterns, mitigate inter-individual variability, and simplify data analysis and interpretation process by focusing on aggregated hemispheric data.

Initially, we explored potential systematic discrepancies between manual delineations and segmentations generated by the deep learning model in the training phase (using the complete Iron dataset). To this end, paired t-tests were applied to assess differences in volume and iron load between manual and automated segmentations within each structure. Additionally, Analysis of Covariance (ANCOVA) was utilised to compare the iron load between manual and automated segmentations, while controlling for volume. These statistical analyses were conducted using the statsmodels library in Python (Seabold & Perktold, 2010).

We conducted partial correlation analyses to evaluate the association between iron load and age, controlling for the effect of volume. These analyses were conducted using the Pingouin library in Python. To assess the correlations between these variables, we utilised Pearson correlation, as implemented in the SciPy library in Python.

Additionally, we used Generalized Additive Models (GAMs) to assess potential nonlinear relationships between iron load and age. GAMs are particularly effective in elucidating relationships between predictors and outcome variables that are not strictly linear, thereby capturing patterns that linear models might overlook. In our analyses, iron load was modelled as the outcome variable, with age incorporated as a smooth term and regional volume as a linear term. We utilised the mgcv package in R to fit, interpret, and visualise the models.

Next, structural equation modelling (SEM) in AMOS (IBM SPSS 26) was used to assess longitudinal changes in regional iron content in the IronAge dataset. To this end, changes were modelled by estimating change regression models, which accommodate the estimation of difference scores independent of the individuals’ initial baseline levels (McArdle, 2009). The model allows to attenuate potential effects pertaining to regression to the mean, a statistical phenomenon, which may be misinterpreted as true change. Regression to the mean may be reflected in strong correlations between baseline and change (Barnett et al., 2005).

We tested whether iron accumulated in the nuclei over time in separate change regression models with continuous age, sex (categorised with women as a reference group), and regional grey-matter volume at baseline for the selected regions as covariates. This model served as a measurement model and defined the relationship between the observed measurements, that is, iron at baseline time-point (TP1) and follow-up time-point (TP2) and the unobserved difference between the time points (TP2–TP1).

We tested the models on two samples: Firstly, a cohort consisting of participants who completed both initial and follow-up MRI sessions, totalling 119 individuals. Secondly, the entire baseline group, comprising 175 participants, which included those who did not return for the follow-up MRI session. Detailed methods for handling missing data, along with the results for the second sample, are provided in theSupplementary Materials. Further, given that regional atrophy (i.e., atrophy of SN, STN, and RN) may increase the concentration of iron and therefore inflate the longitudinal change in iron, we also performed control analyses where a difference score of regional volume (TP2-TP1) was included as an additional covariate together with age, sex, and baseline regional volume.

Statistical outliers for iron in STN (+/-3.29 SD; n = 1), SN, and RN (n = 1) were excluded from analyses and treated as missing (Tabachnick & Fidell, 2014). The following indices were used to evaluate whether the model provided a good representation of the data: the comparative fit index (CFI) and the root mean square error of approximation (RMSEA). Values greater or equal to 0.95 for CFI and values lower or equal to 0.08 for RMSEA were considered to indicate acceptable fit (Kline, 2005). The alpha level for the chi-squared difference test of the models was set to 0.05.

## 3 Results

### 3.1 Performance of deep learning model

#### 3.1.1 Segmentation performance based on input sequences

To select the optimal model concerning combinations of input sequences, we conducted paired t-tests to compare the cross-validated Dice scores across all subjects. Detailed comparisons are presented inTable S1in the Supplementary Material. Briefly, in a single-sequence setup, QSM significantly outperformed T1 and$R_{2}^{*}$(*p*s < 0.001) and performed marginally better than FLAIR (*p*= 0.053). Combining QSM with either T1 or FLAIR significantly improved performance compared to using QSM alone (*p*s < 0.001), except for the combination of QSM and$R_{2}^{*}$(*p*= 0.055). In the multi-sequence setup, models with 3 or 4 sequences did not statistically outperform those combining QSM with either T1 or FLAIR. Thus, we opted for these two-input configurations to lower computational demands.Table 1presents the Dice scores calculated for the segmentation models. For validation, the models were tested on a separate test subset. The results, detailed inTable 2, broadly matched the patterns observed during the training phase.

**Table 1.** The average dice scores and standard deviations of segmentation models trained on different MRI sequences, derived from a five-fold cross-validation process.

**ROI**	**QSM**	**FLAIR**	**T1**	R _2_^*^	**QSM & T1**	QSM & R _2_^*^	**QSM & FLAIR**	**QSM & T1 & FLAIR**	QSM & T1 & R _2_^*^	QSM & FLAIR & R _2_^*^	QSM & FLAIR & T1 & R _2_^*^
**STN-R**	0.78 (0.11)	0.74 (0.02)	0.66 (0.02)	0.70 (0.06)	0.84 (0.02)	0.73 (0.12)	0.85 (0.03)	0.84 (0.03)	0.84 (0.01)	0.85 (0.02)	0.85 (0.02)
**STN-L**	0.78 (0.10)	0.70 (0.03)	0.62 (0.04)	0.66 (0.08)	0.84 (0.02)	0.71 (0.16)	0.84 (0.03)	0.84 (0.03)	0.84 (0.03)	0.84 (0.02)	0.84 (0.02)
**SN-R**	0.85 (0.10)	0.81 (0.01)	0.77 (0.02)	0.77 (0.04)	0.91 (0.01)	0.81 (0.08)	0.91 (0.01)	0.91 (0.01)	0.91 (0.01)	0.91 (0.01)	0.91 (0.01)
**SN-L**	0.85 (0.10)	0.81 (0.01)	0.76 (0.02)	0.75 (0.06)	0.90 (0.01)	0.81 (0.07)	0.90 (0.01)	0.90 (0.01)	0.90 (0.01)	0.91 (0.01)	0.90 (0.01)
**RN-R**	0.88 (0.11)	0.88 (0.01)	0.84 (0.01)	0.85 (0.06)	0.94 (0.01)	0.83 (0.09)	0.94 (0.01)	0.94 (0.01)	0.94 (0.01)	0.94 (0.01)	0.94 (0.01)
**RN-L**	0.87 (0.10)	0.88 (0.01)	0.85 (0.01)	0.83 (0.06)	0.93 (0.01)	0.81 (0.12)	0.93 (0.01)	0.93 (0.01)	0.93 (0.01)	0.94 (0.01)	0.94 (0.01)

STN = subthalamic nucleus; SN = substantia nigra; RN = red nucleus; L = left hemisphere; R: right hemisphere.

**Table 2.** Dice scores of segmentation models evaluated on a test subset of samples, after being trained on different MRI sequences.

**ROI**	**QSM**	**FLAIR**	**T1**	R _2_^*^	**QSM & T1**	QSM & R _2_^*^	**QSM & FLAIR**	**QSM & T1 & FLAIR**	QSM & T1 & R _2_^*^	QSM & FLAIR & R _2_^*^	QSM & FLAIR & T1 & R _2_^*^
**STN-R**	0.84	0.73	0.68	0.73	0.84	0.83	0.85	0.83	0.85	0.85	0.84
**STN-L**	0.85	0.77	0.69	0.77	0.85	0.85	0.85	0.85	0.85	0.85	0.86
**SN-R**	0.91	0.82	0.78	0.83	0.90	0.91	0.91	0.90	0.91	0.91	0.91
**SN-L**	0.91	0.83	0.77	0.81	0.90	0.90	0.90	0.90	0.90	0.90	0.90
**RN-R**	0.94	0.88	0.87	0.88	0.93	0.93	0.93	0.93	0.93	0.93	0.93
**RN-L**	0.93	0.87	0.84	0.87	0.93	0.93	0.93	0.93	0.93	0.93	0.93

STN = subthalamic nucleus; SN = substantia nigra; RN = red nucleus; L = left hemisphere; R: right hemisphere.

**Fig. 2.**
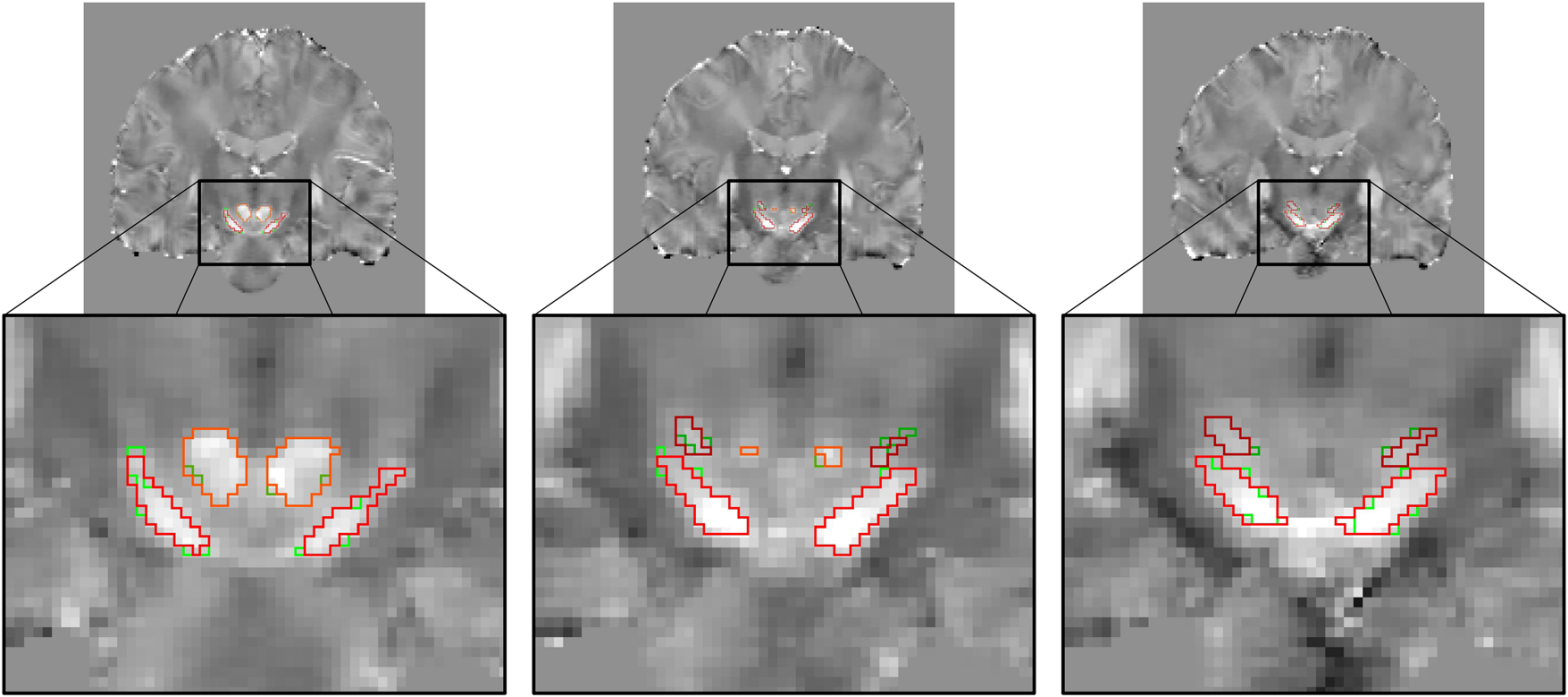
Example of overlap between deep learning model’s segmentation (shown in red contour) and manual tracing (shown in green contour) and of the SN, STN, and RN on coronal slices of a QSM image.

Finally, we retrained the QSM + T1 and QSM + FLAIR models on the complete dataset of 40 subjects from the Iron project to refine their predictive capabilities and resilience. Employing a similar 5-fold cross-validation strategy as in previous sessions, the QSM + FLAIR model achieved Dice coefficients of 0.83, 0.81, 0.90, 0.89, 0.94, and 0.93 for the right and left hemispheres of the STN, SN, and RN, respectively. Comparatively, the QSM + T1 model demonstrated slightly lower performance, with Dice scores of 0.82, 0.82, 0.89, 0.89, 0.92, and 0.91 for the same regions. Although there was no significant performance difference between the models (*p*= 0.228), we selected the QSM + FLAIR model as the final choice due to its higher numerical Dice scores. However, both models have been made publicly available to ensure maximum compatibility with available sequences in the datasets (https://github.com/ffalahati/midbrain_segmentation).Figure 2illustrates an example of the overlap between manual tracings and segmentations generated by the deep learning model.

#### 3.1.2 Manual and automated segmentation agreements

Regarding volumetric comparisons between manual delineations and those from the automated deep learning model, the paired t-tests revealed no significant volumetric differences between manual and deep learning-based segmentations: STN (t(39) = 1.53,*p*= 0.135), SN (t(39) = 0.41,*p*= 0.685), and RN (t(39) = 0.75,*p*= 0.456). Separate paired t-tests conducted on average iron values showed no significant differences for the STN (t(39) = 1.13,*p*= 0.266) and RN (t(39) = 1.48,*p*= 0.146). However, a statistically significant difference was identified in the SN (t(39) = 2.87,*p*= 0.007), with deep learning segmentation exhibiting a slightly higher iron load (mean = 0.152, std = 0.026) compared to manual tracing (mean = 0.151, std = 0.025). Further analysis indicated that this difference was driven by the group of older subjects (t(17) = 2.673,*p*= 0.016), but not by the younger subjects (t(21) = 1.608,*p*= 0.123).

**Fig. 3.**
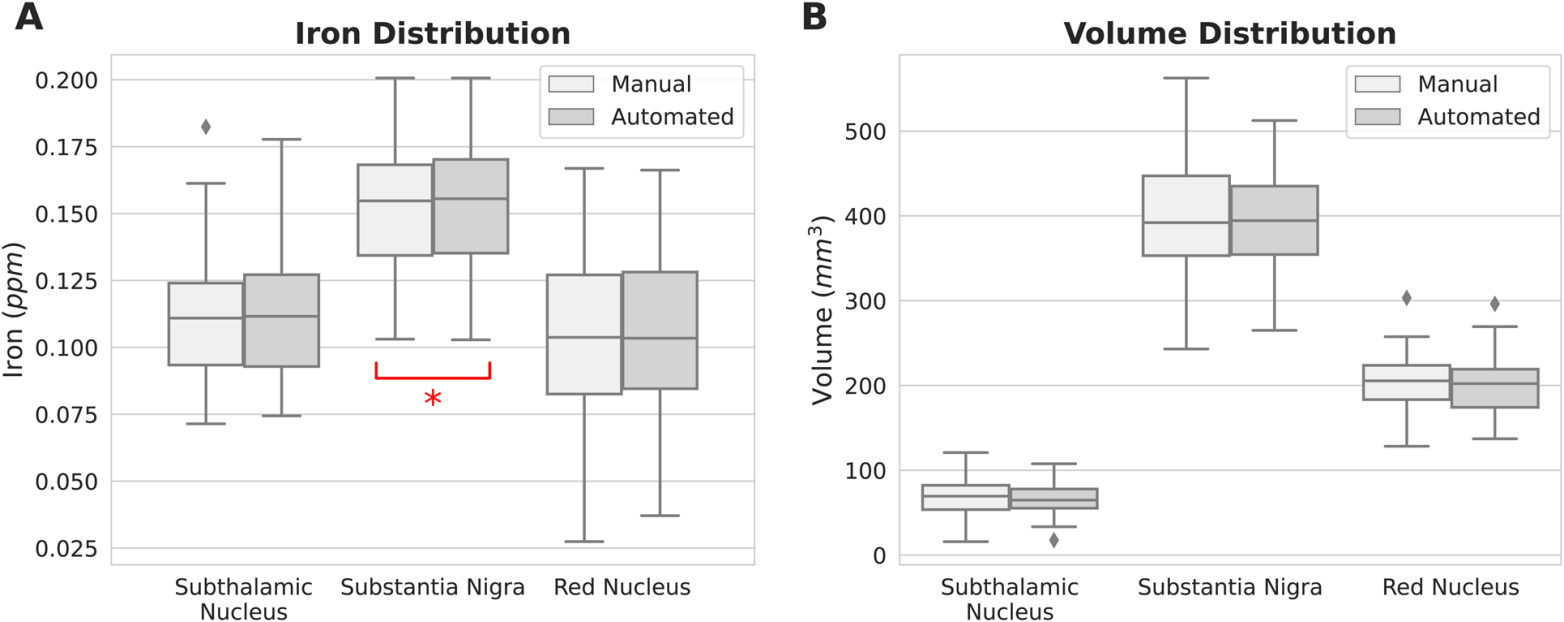
The distribution of (A) iron load and (B) volume, extracted on the QSM images using the manual delineation and automated segmentation labels, stratified by structures. The red asterisk indicates a statistically significant difference between the distributions (*p*= 0.007).

Additionally, we investigated whether the volume of segmented structures could account for the observed differences in iron load between methods within the SN. The ANCOVA results indicated that the segmentation method did not significantly impact the iron load (F(1, 77)= 0.058,*p*= 0.810), suggesting that the differences between methods are not statistically significant when volume is considered. Conversely, volume itself significantly contributed to the variance in iron load (F(1, 77) = 5.08,*p*= 0.027), implying that variations in volume are associated with notable differences in iron levels.Figure 3illustrates the distribution of volume and iron content in each region, as extracted from manually and automatically segmented structures.

The correlations between the manual and automated segmentations for the extracted volumes and iron load were strong, ranging from 0.8 to 0.94 (*p*s < 0.001) for volume, and between 0.96 and 0.99 (*p*s < 0.001) for iron load.

We further examined whether the correspondence between manual and automated segmentation approaches was consistent across different age groups. To this end, we separately compared both volume and iron load between younger and older adults for each segmentation method. The results, as illustrated inFigure 4, indicated that iron load was significantly higher in older individuals compared to younger adults across all three structures. Whereas there was no significant difference in volume between the younger and older age groups in the STN and SN, the volume of the RN was significantly lower in the older group. The patterns of age differences were consistent between manual and automated segmentation approaches.

**Fig. 4.**
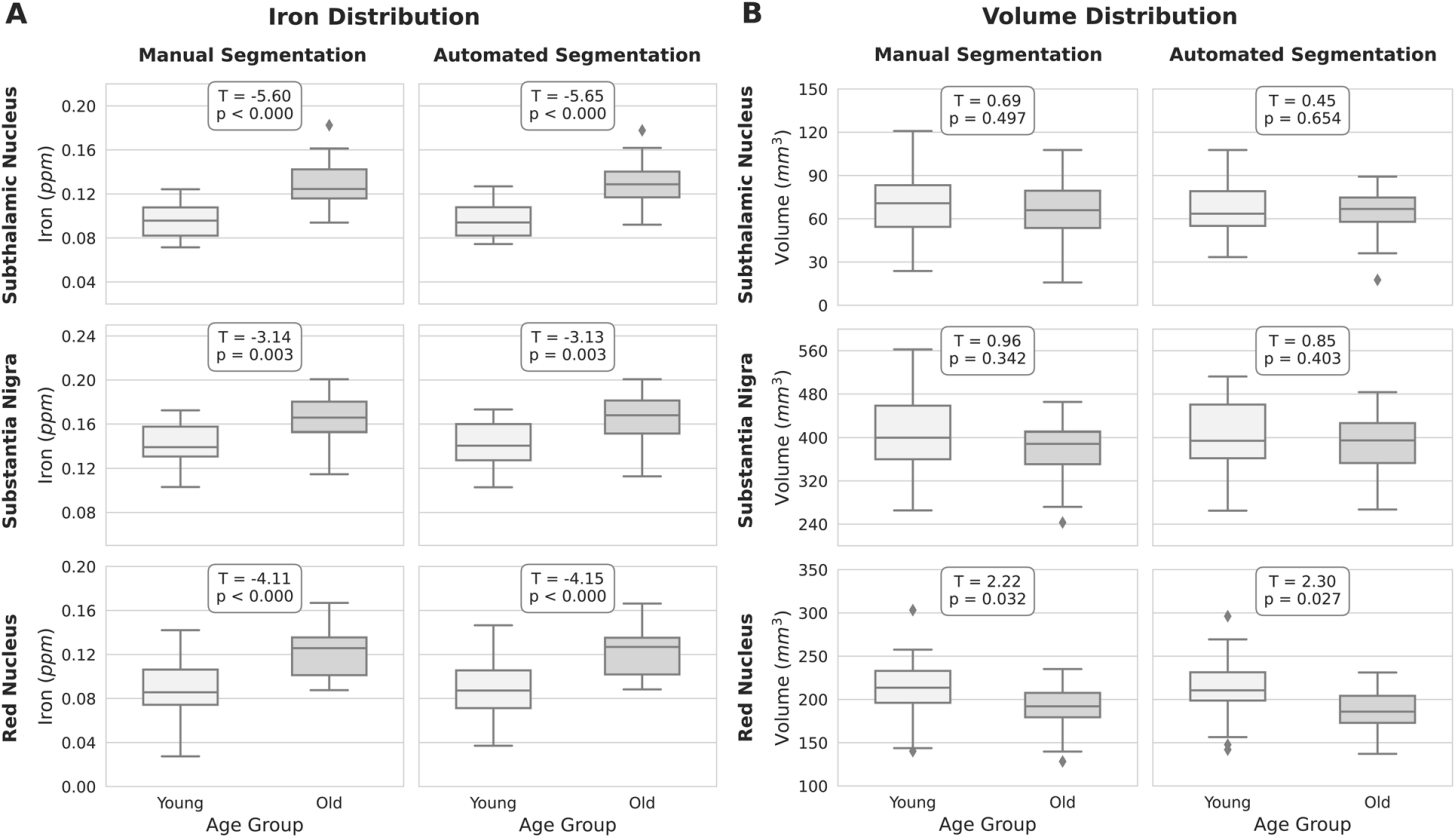
The distribution of (A) iron load and (B) volume, extracted on the QSM images using the manual delineation and automated segmentation labels, compared between the younger and older age groups within Iron (training) dataset. The corresponding t-statistic and*p*-value from the t-test used to compare the variable between the age groups are annotated on each subplot.

### 3.2 Age-related iron changes on the IronAge dataset

The deep learning model, trained on the entire iron cohort, was subsequently applied to the longitudinal IronAge cohort, which consisted of 175 participants at baseline and 134 participants at follow-up, with 119 subjects having both baseline and follow-up data. All generated segmentations underwent visual quality control prior to further analyses. This inspection was performed by two authors, G.K. and J.G., with manual corrections implemented as necessary (G.K.). Out of 309 segmented images, corrections were required in 11 (3.6%) cases, where minor adjustments of the labels for one or more structures were needed. We utilised labels generated by the final trained model to calculate the average iron load across all voxels within each nucleus.

#### 3.2.1 Cross-sectional aging effects

We initially assessed the cross-sectional associations between iron load in each nucleus and age at both baseline and follow-up time points. First, we calculated the associations between iron load and age, volume and age, and iron load and volume independently. Higher iron load was significantly correlated with older age across all nuclei, except for the RN at follow-up, which showed a trend towards significance. Additionally, the correlation analyses indicated an association between iron load and the volume of the structures. These correlations remained significant when controlling for the age of subjects (*p*s ≤ 0.002). Notably, in the RN, there was a significant negative correlation between volume and age at both time points. Next, we investigated whether the association between iron load and age remained significant when controlling for the volume of structures. We conducted partial correlations between iron load and age, controlling for the volume of structures; the age-iron associations remain largely significant. The results are summarised inTable 3.

**Table 3.** Bivariate correlations between iron load and age, volume and age, iron load and volume, and partial correlations between iron load and age controlling for volume, for each nucleus, at baseline (N = 175) and follow-up (N = 134).

**ROI**	**Time point**	**Iron load – Age**		**Volume – Age**		**Iron load – Volume**		**Iron load – Age |Volume**	
		***r***	***p***	***r***	***P***	***r***	***p***	***r***	***p***
**STN**	**Baseline**	0.49	<0.001	0.05	0.508	0.39	<0.001	0.52	<0.001
	**Follow-up**	0.38	<0.001	-0.18	0.038	0.37	<0.001	0.48	<0.001
**SN**	**Baseline**	0.29	<0.001	0.06	0.439	0.24	0.002	0.29	<0.001
	**Follow-up**	0.28	0.008	-0.03	0.778	0.41	<0.001	0.26	0.002
**RN**	**Baseline**	0.35	<0.001	-0.26	0.001	0.21	0.006	0.43	<0.001
	**Follow-up**	0.15	0.076	-0.41	<0.001	0.34	<0.001	0.34	<0.001

STN = subthalamic nucleus; SN = substantia nigra; RN = red nucleus;*r*= Pearson correlation coefficient,*p*=*p*-value.

**Fig. 5.**
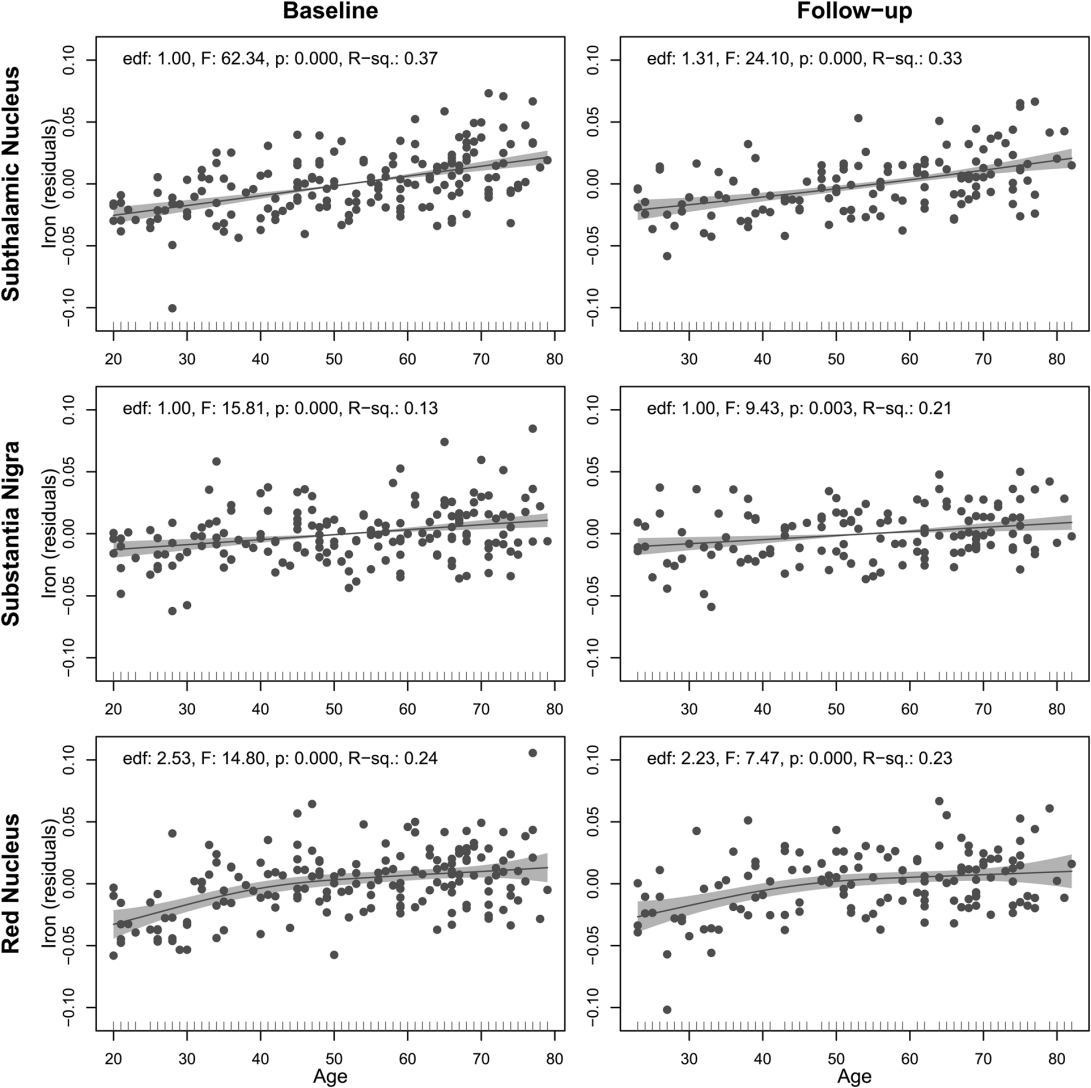
Associations between iron load (residuals, adjusted for volume) and age at baseline and follow-up, stratified by nucleus. Each plot displays a scatter plot with a line representing the smoothed effect of age on iron load residuals, accompanied by 95% confidence intervals (shaded in gray) revealed by GAM analyses. The corresponding effective degrees of freedom (edf), F-statistic, and*p*-value for the smooth term from the GAM model and the adjusted R-squared (R-sq) for the GAM model are annotated on each plot.

Furthermore, we assessed potential nonlinear relationships between iron load and age using GAM analyses. The GAMs for the SN and STN indicated linear relationships between iron load and age at both baseline and follow-up. However, the GAMs for the RN revealed nonlinear relationships. For the baseline analysis, the nonparametric component of the model, representing age, demonstrated significant nonlinearity in its relationship with iron load, evidenced by an effective degrees of freedom (edf) of 2.53, F = 14.8,*p*< 0.001. The adjusted R-squared for this model was 0.24. The analysis on the follow-up data also revealed significant nonlinearity between age and iron load, with an edf of 2.23, F = 0 7.5,*p*< 0.001. The adjusted R-squared for this model was 0.23.Figure 5illustrates the fitted models.

#### 3.2.2 Longitudinal changes in iron

Changes in regional iron were estimated in change regression models using structural equation modelling incorporating age, sex, and baseline volume as covariates, and applied to the IronAge participants who had two MRI sessions (n = 119). SeeFigure 6for results.

**Fig. 6.**
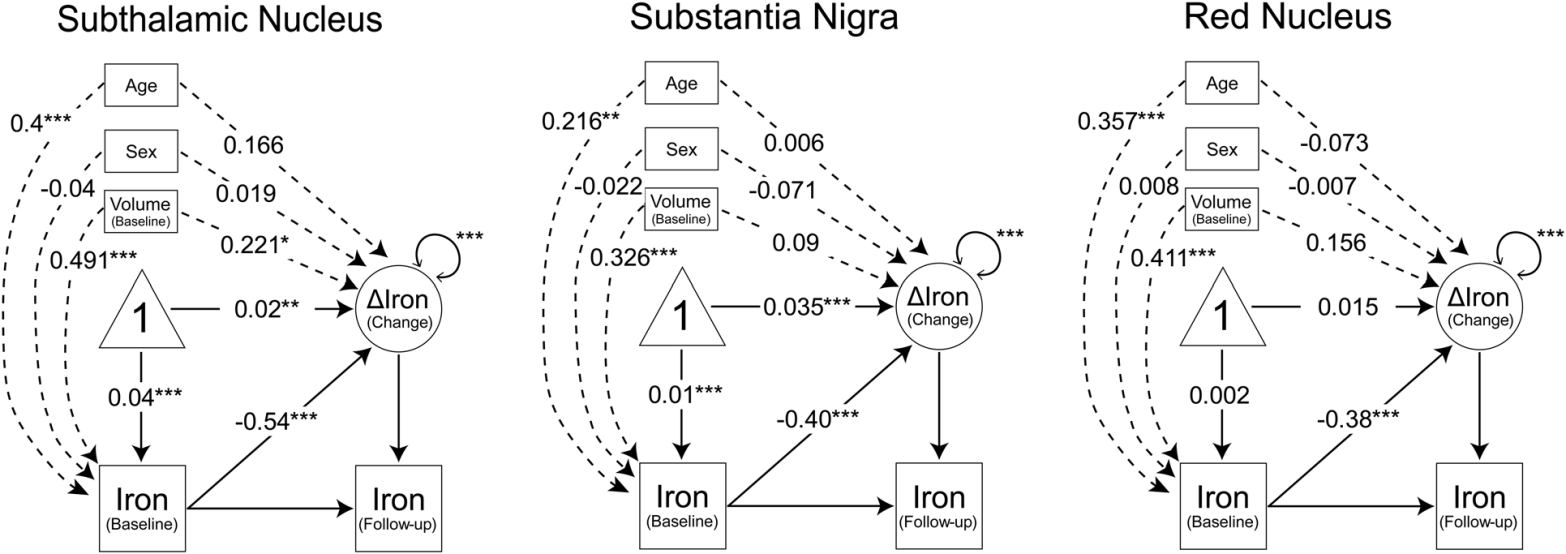
Structural Equation Models for estimating longitudinal iron changes. Measured (observed) variables are represented by rectangles, change (latent) by a circle, and a constant by a triangle. Arrows from constant to baseline and from constant to change represent mean levels at baseline and mean change, respectively. One-headed arrows with numbers represent regressions presented as standardised parameter estimates.

The model for STN exhibited an excellent fit [χ^2^(1, n = 119) = 0.005,*p*= 0.95, CFI = 1.00, RMSEA = 0.00, 90% CI: 0.00-0.035]. There was a significant increase in iron over time (Mean increase = 0.02,*p*= 0.005), and variance in change was significant (*p*< 0.001). Furthermore, more iron at baseline was associated with less iron accumulation (β = −0.541,*p*< 0.001), and older age was associated with more iron at baseline (β = 0.4,*p*< 0.001), but was only marginally associated with higher accumulation of iron (β = 0.166,*p*= 0.08). A higher volume at baseline was associated with more iron at baseline (β = 0.491,*p*< 0.001) and with greater increase in iron (β = 0.221,*p*= 0.027).

The model for SN exhibited an excellent fit [χ^2^(1, n = 119) = 0.005,*p*= 0.95, CFI = 1.00, RMSEA = 0.00, 90% CI: 0.00-0.035]. There was a significant increase in iron over time (Mean increase = 0.035,*p*= 0.001), and variance in change was significant (*p*< 0.001). More iron at baseline was associated with less iron accumulation (β = −0.398,*p*< 0.001). Older age was associated with more iron at baseline (β = 0.216,*p*< 0.011), but was not associated with change in iron (β = 0.006,*p*= 0.9). A higher volume at baseline was associated with more iron at baseline (β = 0.326,*p*< 0.001) but not with change in iron (β = 0.09,*p*= 0.3).

Lastly, the model for RN exhibited an excellent fit [χ^2^(1, n = 119) = 0,*p*= 0.95, CFI = 1.00, RMSEA = 0.00, 90% CI: 0.00-0.35]. In contrast to SN and STN, there was no significant increase in iron over time (Mean increase = 0.015,*p*= 0.2), but there was still significant variance in change (*p*< 0.001). Just as in SN and STN, more iron at baseline was associated with less iron accumulation (β = −0.38,*p*< 0.001), and older age was associated with more iron at baseline (β = 0.357,*p*< 0.001), but was not associated with change in iron (β = -0.073,*p*= 0.4). A higher volume at baseline was associated with more iron at baseline (β = 0.411,*p*< 0.001) but not with change in iron (β = 0.156,*p*= 0.1).

Sex was not associated to either baseline iron or change in iron in all three models (*p*s > 0.5). The results of the SEM models incorporating the full baseline sample (n = 175) exhibited similar patterns. Detailed findings from these models are presented inTables S2 and S3.

#### 3.2.3 Control analyses

For each structure, an alternative SEM model was assessed where the longitudinal change in the volume of the nucleus was added as an additional covariate in the model. The volumetric changes were calculated as the difference between the volume of each nucleus at follow-up and its volume at baseline. All models exhibited acceptable fit for CFI (≥0.95), but poor fit for RMSEA (>0.09) and were therefore considered inappropriate for interpretation.

## 4 Discussion

In this study, we utilised the nnU-Net deep learning framework and MRI images, particularly QSM data, for automated segmentation of STN, SN, and RN. Our results demonstrated strong agreement between the manual and automated segmentation in terms of Dice scores. We observed superior segmentation accuracy when combinations of QSM with either T1 or FLAIR sequences were utilised as inputs. Furthermore, the application of model to a longitudinal dataset revealed that cross-sectionally, older age was linearly associated with higher iron load in SN and STN, while the association was non-linear in the RN. Longitudinally, we observed iron accumulation over time in STN and SN, but not in RN.

### 4.1 Deep-learning model performance

This study’s application of a deep learning framework for segmenting nuclei in the midbrain demonstrated strong potential and a notable advancement over traditional segmentation methods. Our segmentation method yielded high performance with average Dice scores of 0.82 for the STN, 0.9 for the SN, and 0.94 for the RN, considering manual tracing as gold standard. Particularly, when compared to an atlas-based method that we previously applied on the same dataset (Garzón et al., 2018), our approach demonstrated superior accuracy.

This comparison extends to a range of studies using various techniques for midbrain nuclei segmentation. Investigations that utilised atlas-based methods (Haegelen et al., 2013;Plassard et al., 2019;Visser et al., 2016;Xiao et al., 2012) or traditional machine-learning methods (J. Kim et al., 2018) generally reported inferior results compared to our deep learning approach. This observation is further corroborated byBeliveau et al. (2021),Chai et al. (2022), andZhao et al. (2022). For example, Beliveau et al.’s comparison of CNN architectures to a multi-atlas segmentation model highlighted the superiority of deep learning, with their best models achieving Dice scores that closely align with our findings. Additionally, Chai et al. demonstrated a U-Net-based network outperforming an atlas-based method in segmenting various deep gray matter structures. While direct comparison among these methodologies remains challenging due to varying dataset specifics such as sample size and imaging sequences, the consistency of these results across various studies and methodologies underscores the potential of deep learning as a more accurate tool for neural structure segmentation than traditional methods.

The efficacy of deep learning models, particularly in complex tasks like segmentation of midbrain nuclei, is influenced by a multitude of factors. These include the architecture of the model, the choice of input sequences, and the training samples. In our study, we aimed to provide a comprehensive evaluation of how various input sequences affect model performance when predicting the same labels originally derived from QSM images. In the single-sequence setup, QSM performed better than the other sequence, aligning with the fact that manual labelling was primarily conducted on QSM images due to their superior contrast for the targeted structures. We observed a better performance of FLAIR compared to T1-weighted images, likely due to the better contrast in the targeted structures. This suggests that FLAIR could serve as an alternative MRI modality in scenarios where QSM is not available (see alsoGarzón et al., 2017).

The multi-sequence results showed that utilising a combination of MRI sequences as inputs to the model significantly improved segmentation accuracy. Specifically, integrating information from both QSM and T1-weighted or FLAIR led to an increase in performance. This is in line with previous studies showing that incorporating multiple MR modalities leads to more accurate segmentations. This improvement can be attributed to the complementary nature of these imaging sequences (Hua et al., 2020;W. Zhang et al., 2015). QSM, known for its sensitivity to paramagnetic substances like iron, provides unique contrast that is particularly useful in delineating iron-rich structures. On the other hand, T1-weighted and FLAIR images offer detailed anatomical information, highlighting various aspects of brain tissue structure. By combining these sequences, the model gains a more comprehensive view of the brain’s anatomy and composition, leading to a richer set of features for the model to learn from, resulting in a more nuanced and accurate segmentation.

Furthermore, the training dataset plays a crucial role. Whereas a larger dataset provides a more diverse range of examples from which the model can learn better, the U-net architecture in particular has shown great potential in dealing with smaller training samples (Nemoto et al., 2020;Ronneberger et al., 2015). Additionally, data augmentation helps increase the size of training examples and reduce overfitting by introducing random variations to the original data (Nemoto et al., 2021). Nevertheless, the quality of training data and how well it represents the problem space are equally important. It is crucial to acknowledge the necessity for precise and accurate manually segmented labels for model learning and improvement. Expertly curated datasets are essential to train robust deep learning models and ensure their effectiveness in segmenting structures.

In line with this, our investigation into the differences between manual delineations and segmentations generated by deep learning highlights certain aspects of the model’s performance. The absence of significant volumetric differences suggests that the deep learning approach is comparable to manual delineation in terms of capturing the volumes of the nuclei. However, the discovery of variations in average iron values between manual and automated segmentations in the SN, particularly among older subjects, implies that the model tends to select brighter voxels in the SN. Further examination by visually comparing the segmentations revealed in several subjects, an additional selection of voxels by the deep learning model between the SN and STN—areas where the separation is minimal. Given the current spatial resolution, voxels located in this delicate area likely encompass the SN, the STN, and in-between the two structures. This suggests that the 1 mm thickness on the axial plane of QSM images might be a limiting factor. Both manual and automated segmentation processes could benefit from higher spatial resolution, which might also enhance the performance of automated segmentation.

Moreover, our observation that the discrepancies in iron values were no longer significant after adjusting for the volumes of the structures suggests that this variation might, in part, be attributed to the intrinsic iron-dependent contrast of the QSM images, where the boundaries of a nucleus and consequently its volume might be influenced by the iron load of the nucleus. This further emphasises the need to consider volumetric factors when assessing segmentations based on QSM images.

This insight introduces a new dimension in utilising deep learning models for neuroimaging segmentation. Unlike earlier studies, which predominantly focused on the similarity (Dice coefficient) and correlation between manual tracings and automated segmentations to validate the models—a pattern also observed in our findings—our more in-depth analysis hints at a potential inherent bias, an element not thoroughly investigated in previous research. This finding emphasises the need to consider the confounding effect of volumetric measures when assessing segmentations based on QSM images and highlights the necessity of considering the underlying properties of the imaging modalities which can impact both manual and automated segmentation processes.

Applying the deep learning model, originally trained on the iron dataset, to segment nuclei in the IronAge dataset exemplifies the practical utility of such models. This circumvented the need for labor-intensive, costly manual tracing in a larger longitudinal dataset. Despite the identical imaging parameters and image processing steps in both datasets, the necessity for minor corrections in 3.6% of cases after visual quality control highlights the fact that the most advanced deep learning models are not infallible and emphasises the critical role of expert oversight in ensuring the accuracy of AI-driven segmentation tasks.

### 4.2 Age-related iron changes

Iron levels in the brain are known to increase with age (Madden & Merenstein, 2023). However, previous studies have reported a non-uniform distribution of iron changes across the brain noting that certain regions such as deep gray matter nuclei are more affected than others (Hallgren & Sourander, 1958;Zecca et al., 2004,^2005^). In a histological postmortem study,Hallgren and Sourander (1958)conducted a comprehensive quantitative survey of non-heme iron across various brain regions, including the SN and RN, in individuals ranging from infancy to 100 years of age. They found high iron content in the deep gray matter regions, with the highest concentrations in the globus pallidus, followed by the substantia nigra and red nucleus. Their findings suggested a rapid increase in iron load during the first two decades of life across nearly all brain regions, which then slowed down. Notably, the iron content in regions such as the globus pallidus did not markedly increase after 30 years of age, whereas other regions such as the putamen and caudate nucleus continued to show increased iron levels well beyond 50–60 years of age. While they modelled the relationship between iron load and age using exponential equations for several regions, they did not do so for the SN and RN.

Our cross-sectional analyses, covering the age range of 20 to 80 years, revealed that the SN and STN linearly increased with age, whereas the RN exhibited a nonlinear trajectory characterised by an initial increase followed by a plateau after the fifth decade of life. These findings are closely in agreement with those reported byG. Li et al. (2023), who reported, in 220 healthy individuals aged 10 to 70 years, linear age-related trends in the SN and a nonlinear pattern in the RN, where mean susceptibility values surged before the curve flattened in individuals older than 60 years. Similarly, in a sample of 210 individuals aged 6 to 76 years old,Hagemeier et al. (2013)also reported where a linear association with age in the SN and a nonlinear effect in the RN where a shift in phase images reflecting iron load was observed at around 50 years old. Although there is a consensus in the literature that iron levels are higher in the STN, SN, and RN among older adults compared to younger adults (Acosta-Cabronero et al., 2016;Betts et al., 2016;Bilgic et al., 2012), the age-related patterns reported across a continuous age range are diverse. Indeed, some studies have found no significant changes in iron content with age in the SN (Haacke et al., 2010;X. Xu et al., 2008), whereas other studies have documented linear (Acosta-Cabronero et al., 2016;Burgetova et al., 2021;Gong et al., 2015;Y. Li et al., 2021) or nonlinear associations (W. Li et al., 2014;Persson et al., 2015;Y. Zhang et al., 2018b). In particular in the RN, both linear (Acosta-Cabronero et al., 2016;Burgetova et al., 2021;Gong et al., 2015;Haacke et al., 2010;Y. Li et al., 2021;X. Xu et al., 2008) and nonlinear (Lao et al., 2023;W. Li et al., 2014;Persson et al., 2015;Y. Zhang et al., 2018b) increases in iron levels with age have been reported.

Nevertheless, the cross-sectional nature of these findings may confound the reported age-related differences due to individual variability. Large variances in brain iron content data, particularly among older individuals, have been observed, suggesting considerable person-to-person variation in iron levels and possibly a wide range of trajectories as they age (Acosta-Cabronero et al., 2016;Madden & Merenstein, 2023). A longitudinal examination of the association between iron load and age offers insightful observations into the dynamics of iron accumulation over time. A novel aspect of this study is the use of SEM to investigate longitudinal changes in iron content, providing a comprehensive and region-specific view of iron changes over time with age. Indeed, significant variations in change observed in our SEM models indicate interindividual differences in iron accumulation. Our SEM models indicated significant iron accumulation over time in the STN and SN, aligning with the cross-sectional observations, which showed a linear increase in iron load with age in the STN and SN. The SEM model for STN revealed a trend towards an age effect on iron accumulation (*p*= 0.08), suggesting a possible acceleration of iron accumulation in older age. Although speculative with the current results, this potential association should be assessed in a larger sample that would ideally include individuals older than 80 years old. For the RN, the SEM model revealed no significant increase in iron over time, which also aligns with our cross-sectional observations in the RN, where iron load approaches a plateau as age advances, showing no further significant changes with increasing age. To the best of our knowledge, only one study has investigated iron accumulation in the SN and RN longitudinally in healthy individuals.J. Li et al. (2021)reported significant age-related iron accumulation in the SN and RN within a longitudinal cohort of 44 individuals aged 50 to 80 years, with approximately 8 years between baseline and follow-up measurements. Their observation of longitudinal changes in the RN may be attributed to the extended period between the two time points, which likely allowed for more pronounced differences in iron loads to emerge. In contrast, the 3-year span in our study may not have been sufficient to capture similar differences.

Additionally, the positive association between baseline volume and baseline iron implies that greater volume at baseline is associated with more iron load. Our findings are consistent with those ofRavanfar et al. (2022), who reported a positive correlation between mean QSM values and volume in the SN of healthy individuals. However, associations between smaller volumes and higher iron estimates have been reported cross-sectionally and longitudinally in regions such as the hippocampus and entorhinal cortex (Rodrigue et al., 2011), caudate and putamen (A. M. Daugherty & Raz, 2016), and striatum (A. M. Daugherty et al., 2015).

Overall, the inconsistencies in findings from different studies regarding age-related iron and volume changes in midbrain nuclei may stem from demographic differences, such as varying age distributions among participants, or methodological differences, including imaging sequences, postprocessing techniques used to estimate brain iron content, and the statistical methods employed to identify patterns (G. Li et al., 2023;J. Li et al., 2021).

Besides aging, sex is another factor that may affect brain iron content in healthy adults. Our results showed no association between sex and either baseline iron levels or changes in iron levels across all three nuclei examined. Only a few studies have investigated sex differences in brain iron load or accumulation in these structures, offering contrasting insights. Consistent with our findings,Xu et al. (2008)andAcosta-Cabronero et al. (2016)reported no significant sex-related differences in iron levels. However,Persson et al. (2015)observed that men had significantly more iron than women in the SN, a trend also noted for the RN. Furthermore,Gong et al. (2015)found more iron in the RN in men than in women. These results emphasise the need for a systematic investigation into the impact of sex on iron load and accumulation in these structures.

### 4.3 Limitations and future research directions

The main limitation arises from using QSM images for both the segmentation of structures and the quantification of their volume and iron load. This dual application may introduce inherent biases where the iron-dependent contrast of QSM images could influence the boundaries of the structures, potentially affecting both the measured volumes and iron loads. Thus, it is crucial to consider volumetric factors when assessing iron contents in QSM-based segmentations. In our analyses of age-related differences and changes in iron load (i.e., cross-sectional and longitudinal), we accounted for these volumetric measures. A promising avenue for future research would be to utilise high-resolution QSM images from MRI scanners with stronger magnetic fields (e.g., 7T). These advanced scanners have demonstrated the ability to display anatomical structures consistent with histology (Deistung et al., 2013), offering potential for more precise segmentation and quantification of iron load and volume.

In this study, we explored age-related changes in iron using data from only two time points, restricting our ability to fully model non-linear trajectories of iron accumulation, as such patterns usually require a more extensive dataset with multiple observations over a longer period. Future research, benefiting from more frequent longitudinal measurements, would be more aptly equipped to apply non-linear models and accurately capture the dynamics of iron accumulation in the brain throughout the lifespan.

Another limitation associated with susceptibility mapping is that QSM provides relative susceptibility values, which may not be directly comparable across subjects within a cohort. To address this, different studies have adopted various approaches. While some have opted for absolute susceptibility values, there is a general consensus on referencing these values against a control region, though the choice of this region remains a topic of debate (Madden & Merenstein, 2023). For instance, cerebrospinal fluid (CSF) and various white matter areas have been used, each with its pros and cons (Ravanfar et al., 2021). This decision is important in aging studies where tissue segmentation and age-related changes in tissue properties can influence the reference susceptibility values. For example, incorrectly including iron-rich choroid plexus in CSF segmentations can result in inaccurate reference values (Deistung et al., 2017). Although the least inter-subject variability was reported in the frontal white matter (Deistung et al., 2013), age-related changes such as demyelination or the presence of white matter hyperintensities may skew these reference values. Nevertheless, previous studies have reported that the method of referencing does not alter the detection of age-related differences in QSM values (Acosta-Cabronero et al., 2016;W. Li et al., 2014). In this study, we used a white matter region centered at MNI coordinates [−24; −27; 39] to maintain methodological consistency between training and test datasets. It is important to note, however, that while these coordinates were chosen based on a region with minimal R2* variability in a previous study, such consistency might not extend across different cohorts.

Another limitation of the current study is the lack of comparisons for additional core nuclei known to accumulate iron with aging, such as those within the basal ganglia. While our study focused on the STN, SN, and RN, expanding such analyses by including other regions could enhance the generalisability and robustness of our findings, as well as further validate our segmentation methods. However, the absence of manual tracings for these regions in our dataset restricted our ability to conduct a broader comparative investigation. Including a more diverse array of brain regions in model training processes in future studies would not only facilitate comprehensive validation of segmentation methods but also enable a more detailed exploration of iron deposition throughout the brain.

Looking ahead, it is important to explore and validate the use of our segmentation method in other cohorts, especially those with different imaging parameters. This would not only help in generalising the applicability of our method but also in understanding the nuances of iron accumulation across diverse populations. Increased iron accumulation in the SN in PD patients has been reported previously (Ulla et al., 2013;Ward et al., 2014;Zecca et al., 2004), and abnormal iron deposition in the SN has been suggested as a potential biomarker for helping in diagnosis PD patients (E. Y. Kim et al., 2018) and assessing disease severity (Ghassaban et al., 2019). These findings underscore the potential of applying our deep learning segmentation method to datasets of PD patients.

## 5 Conclusion

In this study, we effectively employed a deep learning framework, specifically nnU-Net, for the automated segmentation of the STN, SN, and RN. Our findings reveal superior segmentation accuracy, achieved particularly through the integration of structural MRI and magnetic susceptibility maps. Our detailed analysis of the agreement between manual and automated segmentation indicates a potential interplay between volume and iron load, underscoring the importance of considering the confounding effect of volumetric measures in assessments of iron load in the segmented structures. Furthermore, applying the automated segmentation method to a longitudinal dataset has provided novel insights into the patterns of iron accumulation over time in the midbrain nuclei, with significant increases observed in the STN and SN, but not in the RN.

Our findings shed light on normal aging-associated iron accumulation patterns in these nuclei. Further research in the context of neurodegenerative diseases, where such iron deposition may play a critical role, could be instrumental in understanding the implications of these patterns. Future efforts will aim to broaden the framework’s applicability across images from various MRI scanners and to investigate its effectiveness in larger and more diverse cohorts, including those with neurodegenerative conditions. By making our trained networks publicly available, we seek to support wider research initiatives and promote the use of sophisticated computational techniques in neurological research.

## Supplementary Material

Supplementary Material

## Data Availability

The codes and trained deep learning networks are available athttps://github.com/ffalahati/midbrain_segmentation. The raw data cannot be made publicly available because the disclosure of personal data was not included in the research protocol and informed consent documents. However, access to the raw data may be available upon reasonable request to the Principal Investigator of the Iron and IronAge projects, G.K.,gregoria.kalpouzos@ki.se.
