# Supplementary material for: Automated segmentation of midbrain nuclei using deep learning and multisequence MRI: A longitudinal study on iron accumulation with age
Source: Imaging Neurosci (Camb). 2024 Oct 7;2:imag-2-00304. doi: 10.1162/imag_a_00304 (PMC12290624; doi:10.1162/imag_a_00304)
Supplement: Supplementary Material [file imag_a_00304-supp.pdf]

Supplementary Materials

S1. Comparison of model performance with regard to input sequences

To identify the most effective combinations of input sequences for optimal segmentation performance, we conducted paired t-tests to analyze the cross-validated Dice scores from all subjects. Table S1 shows the outcomes of these comparisons, including t-statistics and p-values.

Table S1. Comparison of cross-validated dice scores across input sequences.

Input Sequence(s) 1	Input Sequence(s) 2	T-Statistic	P-Value
QSM	FLAIR	1.949	0.053
QSM	T1	5.265	< 0.001
QSM	R_2*	3.613	< 0.001
QSM	QSM-T1	-3.779	< 0.001
QSM	QSM-R_2*	1.932	0.055
QSM	QSM-FLAIR	-3.768	< 0.001
QSM	QSM-T1-R_2*	-3.667	< 0.001
QSM	QSM-T1-FLAIR	-3.649	< 0.001
QSM	QSM-FLAIR-R_2*	-3.866	< 0.001
QSM	QSM-FLAIR-T1-R_2*	-3.714	< 0.001
FLAIR	T1	11.411	< 0.001
FLAIR	R_2*	3.447	0.001
FLAIR	QSM-T1	-23.271	< 0.001
FLAIR	QSM-R_2*	0.957	0.34
FLAIR	QSM-FLAIR	-21.933	< 0.001
FLAIR	QSM-T1-R_2*	-23.988	< 0.001
FLAIR	QSM-T1-FLAIR	-22.753	< 0.001
FLAIR	QSM-FLAIR-R_2*	-24.318	< 0.001
FLAIR	QSM-FLAIR-T1-R_2*	-24.389	< 0.001
T1	R_2*	-0.61	0.542
T1	QSM-T1	-25.432	< 0.001
T1	QSM-R_2*	-1.566	0.119
T1	QSM-FLAIR	-24.868	< 0.001
T1	QSM-T1-R_2*	-25.202	< 0.001
T1	QSM-T1-FLAIR	-25.13	< 0.001
T1	QSM-FLAIR-R_2*	-25.521	< 0.001
T1	QSM-FLAIR-T1-R_2*	-25.048	< 0.001
R_2*	QSM-T1	-10.233	< 0.001
R_2*	QSM-R_2*	-1.297	0.196
R_2*	QSM-FLAIR	-10.096	< 0.001
R_2*	QSM-T1-R_2*	-10.205	< 0.001
R_2*	QSM-T1-FLAIR	-9.962	< 0.001
R_2*	QSM-FLAIR-R_2*	-10.23	< 0.001
R_2*	QSM-FLAIR-T1-R_2*	-10.203	< 0.001
QSM-T1	QSM-R_2*	5.407	< 0.001
QSM-T1	QSM-FLAIR	-0.235	0.815
QSM-T1	QSM-T1-R_2*	0.921	0.358
QSM-T1	QSM-T1-FLAIR	1.711	0.089
QSM-T1	QSM-FLAIR-R_2*	-1.829	0.069
QSM-T1	QSM-FLAIR-T1-R_2*	0.232	0.817

QSM-R_2*	QSM-FLAIR	-5.438	< 0.001
QSM-R_2*	QSM-T1-R_2*	-5.378	< 0.001
QSM-R_2*	QSM-T1-FLAIR	-5.306	< 0.001
QSM-R_2*	QSM-FLAIR-R_2*	-5.507	< 0.001
QSM-R_2*	QSM-FLAIR-T1-R_2*	-5.397	< 0.001
QSM-FLAIR	QSM-T1-R_2*	0.778	0.437
QSM-FLAIR	QSM-T1-FLAIR	1.764	0.079
QSM-FLAIR	QSM-FLAIR-R_2*	-1.499	0.135
QSM-FLAIR	QSM-FLAIR-T1-R_2*	0.378	0.706
QSM-T1-R_2*	QSM-T1-FLAIR	0.733	0.464
QSM-T1-R_2*	QSM-FLAIR-R_2*	-2.803	0.006
QSM-T1-R_2*	QSM-FLAIR-T1-R_2*	-0.599	0.55
QSM-T1-FLAIR	QSM-FLAIR-R_2*	-3.127	0.002
QSM-T1-FLAIR	QSM-FLAIR-T1-R_2*	-1.322	0.188
QSM-FLAIR-R_2*	QSM-FLAIR-T1-R_2*	2.043	0.042

A positive T-statistic indicates that the average Dice score of the first sequence(s) is higher than that of the second sequence(s), meaning the first sequence(s) performs better. Conversely, a negative T-statistic indicates a higher performance of the second sequence(s).

S2. Longitudinal changes in iron without adjusting for volume

Changes in regional iron of the nuclei were estimated in change regression models using structural equation modelling with age and sex as covariates. The data is reported in full detail in Figure S1. All three models exhibited excellent fit [χ2 (1, n = 119) = 0.005, $p > 0.9$, CFI = 1.00, RMSEA = 0.00 90% CI: 0.00-0.035]. Iron increased significantly in all three nuclei over time and the variance in change was significant as well ($p < 0.001$), indicating interindividual differences in change. Furthermore, baseline iron was negatively associated with iron accumulation in all nuclei, suggesting that more baseline iron was related to less increase. Older age was associated with more iron at baseline, but not with change in iron. Sex was not associated to neither baseline iron nor iron change for any of the nuclei.

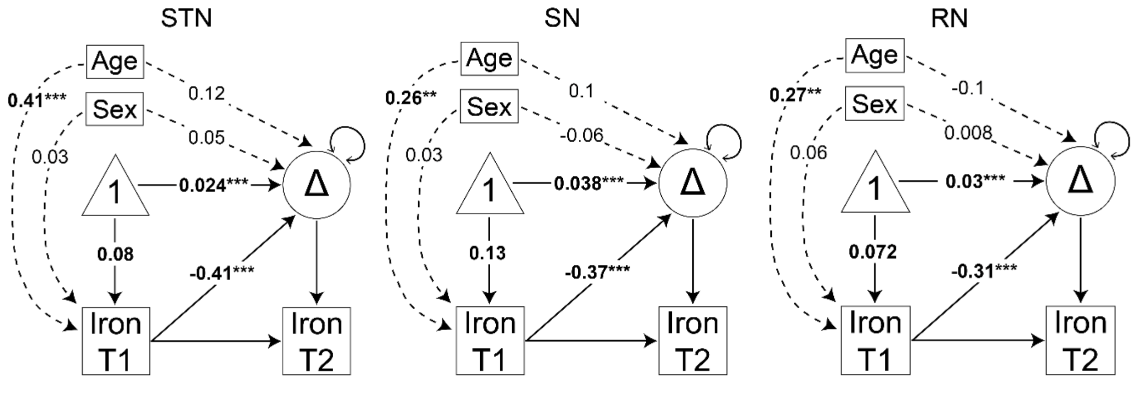

Figure S1. *Measured (observed) variables are represented by rectangles, change (latent) by a circle, and a constant by a triangle. Arrows from constant to baseline and from constant to change represent mean levels at baseline and mean change, respectively. One-headed arrows with numbers represent regressions presented as standardised parameter estimates. T1 = baseline; T2 = Follow-up; Δ = change; SN = substantia nigra; STN = subthalamic nucleus; RN = red nucleus.*

S3. Longitudinal changes in iron in full dataset

Models' results for all regions using the full sample at baseline are reported in Table S2 (without adjusting for volume) and Table S3 (adjusted for volume). To accommodate for missing data due to the attrition between time point 1 and 2, full information maximum likelihood (FIML) estimates (Finkbeiner, 1979; Schafer and Graham, 2002) were used for all models. FIML is a procedure which uses available information for estimating parameters that contain variables with missing values rather than imputing or omitting data. Using FIML allows for a more accurate population estimate and a better measurement model compared to other procedures dealing with missing data, such as listwise deletion (Schafer and Graham, 2002). The estimates are unbiased under the assumption of missing at random. That is, the likelihood of data from a variable missing may depend on other variables in the model rather than the variable itself (Little and Rubin, 2002). In our sample, only age was a significantly differentiating variable with dropouts being younger than returnees (t(206) = -2.1, *p* = 0.03), whereas other variables such as iron content were not. To support the assumption of missing at random (i.e., that the missingness can be explained by observed variables at baseline), we included predictors such as chronological age, and sex in our models to account for the largest part of the missing data (Rubin, 1975; Schafer and Graham, 2002). The data introduced by these covariates are included in the variance-covariance matrix that is used to optimize the likelihood of the model parameter values estimated by FIML, to be accurate and unbiased when missing at random (Staudt et al., 2022).

Table S2. Standardized beta coefficients onto respective regional iron measurement in full sample (n = 175). Models are adjusted for age and sex.

	Subthalamic Nucleus			Substantia Nigra			Red Nucleus		
	BSL Iron	Δ Iron		BSL Iron	Δ Iron		BSL Iron	Δ Iron	
**Age**	0.486***	0.125		0.276***	0.012		0.336***	-0.128	
**Sex**	-0.046	0.05		-0.078	-0.055		-0.011	0.008	
**BSL Iron**		-0.426***			-0.389***			-0.325***	
**Mean Iron change**			0.024***			0.038***			0.030***

*BSL = Baseline; Δ = Change; *p < 0.05; **p < 0.01; ***p < 0.001.*

Table S3. Standardized beta coefficients onto respective regional iron measurement in full sample (n = 175). Models are adjusted for age, sex, and regional volume.

	Subthalamic Nucleus			Substantia Nigra			Red Nucleus		
	BSL iron	Δ iron		BSL iron	Δ iron		BSL iron	Δ iron	
**Age**	0,447***	0,172		0,247***	0,007		0,411***	-0,076	
**Sex**	-0,081	0,019		-0,106	-0,07		-0,046	-0,007	
**BSL volume**	0,41***	0,224*		0,234***	0,099		0,386***	0,159	
**BSL iron**		-0,556***			-0,419***			-0,392***	
**Mean iron change**			0,020**			0,035**			0.015

*BSL = Baseline; Δ = Change; *p < 0.05; **p < 0.01; ***p < 0.001.*

S4. References

Finkbeiner, C., 1979. Estimation for the multiple factor model when data are missing. Psychometrika 44, 409–420. https://doi.org/10.1007/BF02296204

Little, R.J.A., Rubin, D.B., 2002. Statistical Analysis with Missing Data, 1st ed, Wiley Series in Probability and Statistics. Wiley. https://doi.org/10.1002/9781119013563

Rubin, D.B., 1975. Inference and Missing Data. ETS Res. Bull. Ser. 1975, i–19. https://doi.org/10.1002/j.2333-8504.1975.tb01053.x

Schafer, J.L., Graham, J.W., 2002. Missing data: Our view of the state of the art. Psychol. Methods 7, 147–177. https://doi.org/10.1037/1082-989X.7.2.147

Staudt, A., Freyer-Adam, J., Ittermann, T., Meyer, C., Bischof, G., John, U., Baumann, S., 2022. Sensitivity analyses for data missing at random versus missing not at random using latent growth modelling: a practical guide for randomised controlled trials. BMC Med. Res. Methodol. 22, 250. https://doi.org/10.1186/s12874-022-01727-1
